# Small bowel perforation caused by pancreaticojejunal anastomotic stent migration after pancreaticoduodenectomy: A case report

**DOI:** 10.1097/MD.0000000000021120

**Published:** 2020-07-24

**Authors:** Li Bao, Zhi-Tao Chen, Jia-Cheng Huang, Meng-Xia Li, Le-Le Zhang, Da-Long Wan, Sheng-Zhang Lin

**Affiliations:** a Department of Hepatobiliary and Pancreatic Surgery, First Affiliated Hospital, Zhejiang University School of Medicine; b Zhejiang University School of Medicine, Hangzhou, China.

**Keywords:** bowel perforation, case report, pancreatic fistula, pancreaticoduodenectomy, pancreaticojejunal anastomotic stent, stent migration

## Abstract

**Introduction:**

Pancreaticoduodenectomy (PD) has been widely applied as a standard surgical procedure to treat periampullary diseases. The placement of a pancreaticojejunal anastomotic stent is considered an effective and safe method for preventing pancreatic fistula after PD. Recently, the role of pancreaticojejunal anastomotic stents has been challenged, as gradually increasing complications have been observed. Stent-related small bowel perforation has only occurred in 2 cases as long-term complications but has not been reported to occur within 1 week after surgery.

**Patient concerns:**

Here, we report the case of a 71-year-old female patient complaining of painless jaundice who underwent PD with a pancreaticojejunal anastomotic stent for a duodenal papillary adenocarcinoma (T4N1M0). Four days after surgery, she had a sudden rise in temperature, high white blood cell count, significantly elevated C-reactive protein and 400 ml green-brown drainage fluid. Enhanced computed tomography showed hydrops abdominis.

**Diagnosis:**

Small bowel perforation caused by stent migration was considered first.

**Interventions:**

An emergency exploratory laparotomy was performed. We located the pancreaticojejunal anastomotic stent, which extended 2 cm from the small bowel, and sutured the jejunum hole after cutting away the protruding part of the stent.

**Outcomes:**

The patient recovered smoothly and was discharged on the 7th day after the second surgery. After more than 12 months of follow-up, the patient is doing well and is free of any symptoms related to the procedure.

**Conclusion:**

We caution that stent-related complications can occur when perioperative patients suffer from unexplained or sudden changes in vital signs after PD. In addition, the function of the pancreaticojejunal anastomotic stent needs to be reevaluated by future studies.

## 1 Introduction

With the continuous improvement of surgical technology, pancreaticoduodenectomy (PD) is regarded as a standard surgical procedure and has been widely applied to treat the periampullary diseases. Currently, the mortality rate of PD has decreased to less than 5%, but pancreatic fistula remains one of the major complications and an important cause of morbidity and mortality after PD, occurring in approximately 10% to 20% of all cases, even in high-volume centers.^[1]^

Therefore, several methods have been explored to reduce the pancreatic fistula rates. The placement of a pancreaticojejunal anastomotic stent is considered an effective and safe method for preventing pancreatic fistula post-PD. Moreover, various stent-induced complications (both short-and long-term) have been reported and receive much attention, including bile duct strictures and stones, liver abscess, stent occlusion or migration, pancreatitis, intestinal obstruction, and intestinal perforation.^[2–6]^ To the best of our knowledge, the long-term complication of stent-related small bowel perforation has occurred in only 2 cases: 1 case occurred 2 years after PD, and the other occurred 19 years after PD.^[4,5]^ However, stent-related small bowel perforation occurring within 1 week after surgery has not been reported before. Recently, we encountered an extremely rare case of a small bowel perforation following PD and successfully managed this complication. Herein, we present the clinical course of the case and our management approaches. The clinical significance, underlying causes, and current views on pancreaticojejunal anastomotic stents will also be discussed briefly.

## 2 Case report

**Figure 1:**
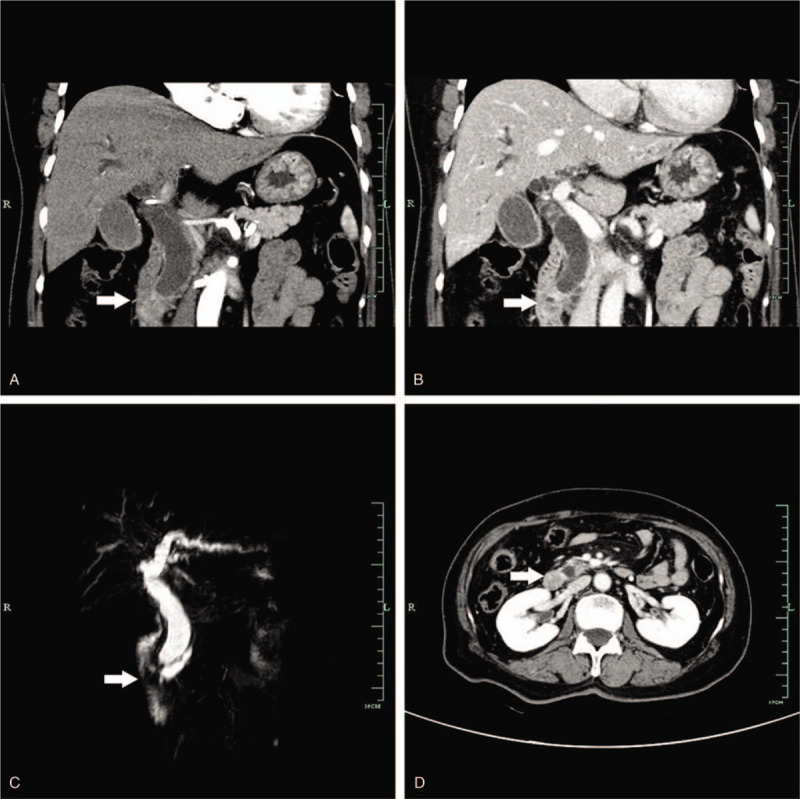
Enhanced CT of the abdomen and MRCP. Enhanced CT of the abdomen showed a dilated common bile duct and a moderately enhanced mass (arrow, 3 × 3 cm) in the area of the duodenal papilla (A-B: coronal view, D: horizontal view). MRCP identified a duodenal papilla mass with corresponding dilated common biliary tracts and main pancreatic ducts (C). CT = computed tomography, MRCP = magnetic resonance cholangiopancreatography.

A 71-year-old Chinese female came to our hospital with complaints of painless jaundice. She had no family history of cancer and her past medical history was negative, except for appendectomy for acute appendicitis 35 years ago. On admission, the physical examination showed moderately icteric sclera and jaundice. The rest of her examination was unremarkable. Laboratory studies revealed the following: elevated total bilirubin 145 μmol/L (normal, 0–21 μmol/L); direct bilirubin 103 μmol/L (normal, 0–5 μmol/L); alkaline phosphatase 253 U/L (normal, 40–150 U/L); aspartate aminotransferase 415 U/L (normal, 5–35 U/L); aspartate transaminase 285 U/L (normal, 8–40 U/L); and carbohydrate antigen 199 78.0 U/mL (normal, 0–37 U/mL). An enhanced computed tomography (CT) scan of the abdomen revealed a dilated common bile duct and pancreatic duct and a moderately enhanced duodenal papilla tumor with a size of 3 × 3 cm (Fig. 1A, B, and D). On magnetic resonance cholangiopancreatography, the common biliary tracts and main pancreatic ducts were markedly dilated due to an obstruction (Fig. 1C). Endoscopic retrograde cholangiopancreatography revealed severe stenosis at the distal common bile duct and a cauliflower-like tumor after cutting the duodenal papilla. Brush cytology of the biliary duct at the time of endoscopic retrograde cholangiopancreatography showed a small cluster of atypical cells. With a tentative diagnosis of duodenal papillary carcinoma, the patient underwent PD.

The gallbladder, distal common bile duct, head of the pancreas, duodenum, distal half of the stomach, lymph nodes around the hepatoduodenal ligament and the common hepatic artery were removed en bloc. Frozen sections revealed duodenal papillary carcinoma, and the resected margins of the biliary tracts and pancreas were free of atypical cells. Reconstruction was performed by the modified Child method. The pancreaticojejunostomy was constructed by the modified Blumgart technique. First, we used 3 double-armed 4–0 polypropylene sutures to place a U-suture with both arms through the pancreatic stump and a 10 to 15 mm longitudinal suture through the seromuscular layer of the jejunum. Subsequently, 5 duct-to-mucosa sutures (1,3,6,9,11 o’clock) were placed using a small single-armed 5–0 polypropylene suture needle with a 15 cm free-floating Nelaton 7 Fr stent in the Wirsung duct. Then, sutures were placed through the seromuscular layer of the jejunum 5 to 7 mm lateral to the previous sutures. These sutures were tied at the ventral wall of the jejunum to completely cover the pancreatic stump with jejunal serosa. Approximately 5 cm further on the jejunal loop, we performed an end-to-side hepaticojejunostomy with continuous absorbable polypropylene (5/0) sutures. After closing the hole in the transverse mesentery, a side-to-side anastomosis was performed for antecolic gastrojejunostomy 55 cm further down on the jejunal loop. The operation time was 434 minutes, and the estimated blood loss was 260 mL. The patient was transferred to the intensive care unit after surgery. On postoperative day 1, she was transferred back to our department without obvious discomfort. However, on postoperative day 4, she had a sudden rise in temperature. The unobstructed posterior drainage tube of pancreaticointestinal anastomosis yielded 400 ml green-brown fluid. Laboratory tests showed a white blood cell count of 16 × 10^9^/L, neutrophils of 91.6%, hemoglobin of 116 g/L, and C-reactive protein of 134.4 mg/L.

**Figure 2:**
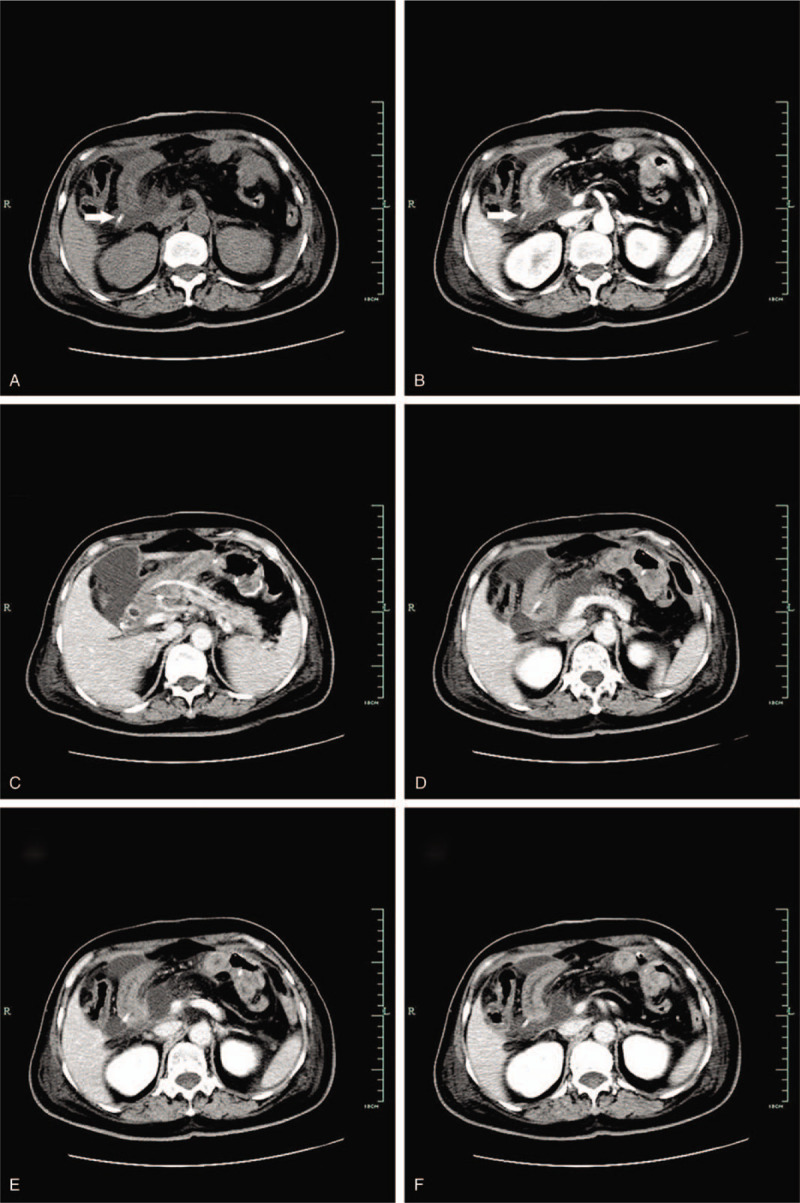
Abdominal enhanced CT 4 days after pancreaticoduodenectomy showed hydrops abdominis, stent (arrow) migration, and bowel limb perforation (A: precontrast CT scan, B: arterial phase, C-F: venous phase). CT = computed tomography.

The enhanced CT showed hydrops abdominis, and small bowel perforation caused by stent migration was considered first (Fig. 2). She underwent an emergency exploratory laparotomy. Intraoperatively, after washing the abdominal cavity, we located the stent, which had perforated the biliary-jejunum limb 3 cm away from the biliary-enteric anastomosis. The stent extended 2 cm from the small bowel. There were no obvious anastomotic leakages from the 3 anastomoses. Therefore, we decided to close the jejunum hole using interrupted absorbable polypropylene 4–0 sutures after cutting away the protruding part of the stent. An abdominal double cannula was placed for repeated lavage if the perforation was not healed. The patient recovered smoothly and was discharged on the 7th day after the second surgery. Histological examination of the surgical specimen revealed moderately and poorly differentiated duodenal papillary adenocarcinoma (T4N1M0), according to the American Joint Committee on Cancer (AJCC) TNM classification.

**Figure 3:**
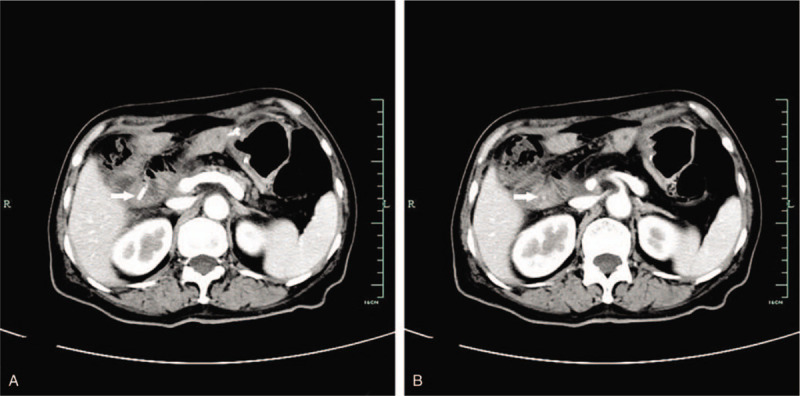
Abdominal enhanced computed tomography 6 months after surgery showed that the residual stent (arrow) remained inside the bowel lumen without other anomalies.

Currently, after more than 12 months of follow up, the patient is doing well and is free of any symptoms related to the procedure (Fig. 3).

## 3 Discussion

Pancreatic fistula is the most common and potentially the most serious complication of PD. Pancreaticojejunal anastomotic stents are widely used to reduce the pancreatic fistula rate, and could provide inner support to the anastomosis and drain the pancreatic juice from the anastomosis, especially in cases with small pancreatic ducts. With the increasing use of pancreatic stents, various stent-related complications have been reported.^[2–6]^

For pancreaticojejunostomy with an external stent, postoperative complications, such as pancreatic fistula, pancreatitis or late-onset stenosis, may occur during or after the stent removal. In contrast, it is generally thought that the internal stent usually falls off spontaneously and is expelled through defecation. Kadowaki et al performed a retrospective study and determined that the median time from placement to being expelled through defecation and the cumulative defecation expulsion rate at 1 year were 454 days and 41%, respectively.^[3]^ The incidence of internal stent migration into the bile ducts and stent-induced complications were 16.8% and 29.6%, respectively, in a previous study.^[6]^ In previous studies, it has been proven that delayed detachment and migration are the 2 main causes of complications after pancreaticojejunostomy with an internal stent.^[3,6]^ Stent-related complications often occur within 1 to 2 years postoperatively (range of 6 weeks to 19 years), and perioperative complications that require surgical operation secondary to stent migration are very rare.^[3,4]^ To the best of our knowledge, stent-related bowel perforation occurring within 1 week after surgery has not been reported before. In our case, the patient promptly underwent surgical treatment owing to the typical clinical and imaging manifestation of intestinal perforation. However, the triggers that led to stent migration and caused the bowel perforation remain relatively obscure and untraceable. We thought there may be three reasons: the first reason is that the pancreatic stent was too long. As a foreign matter, the stent has a harder texture and is easily colonized by bacteria. The second is that the intestinal wall was edematous. Finally, we doubted that the transcolonic mesentery hole was too tight to close and the proximal jejunum was so short that it twisted into angles. Stents stimulated the edematous wall of the bowel along with peristalsis and finally led to the perforation. Nonetheless, a more optimized explanation with compelling evidence is still needed. Fortunately, we detected the problem in time and corrected it in an efficient way. In addition, the patient had no postoperative pancreatic leakage, so we simply placed interrupted sutures to close the perforation. If we misdiagnosed the intestinal fistula as a bile leak, abdominal infection could have led to pancreatic fistula, and then retroperitoneal bleeding would have caused irreparable consequences. We advise that emergency CT is necessary if patients have a sudden rise in temperature after PD.

Presently, the role of pancreaticojejunal anastomotic stents has been challenged because of the gradually increasing incidence of complications. There is a question worth deep consideration: is it necessary to place a pancreaticojejunal anastomotic stent? First, several studies demonstrated that PD without pancreaticojejunal anastomotic stent is safe and reliable and has no significant differences from that with a stent in the incidence of pancreatic fistula. Second, stent placement will increase the operating difficulty and require a longer surgical time. Finally, stent-related complications are easily overlooked in disease management and are associated with increased morbidity and mortality, and prolonged hospital stays.^[7–10]^

In conclusion, although pancreatic stenting during pancreatoduodenectomy is generally considered to be a safe method for preventing pancreatic fistula, this approach is not free from potential complications. Stent-related complications should be considered when perioperative patients suffer from unexplained or sudden changes in vital sighs after PD. In addition, the function of the pancreaticojejunal anastomotic stent needs to be reevaluated by future studies.

## Author contributions

**Supervision:** ShengZhang Lin.

**Validation:** ShengZhang Lin.

**Writing – original draft:** Li Bao, Zhi-Tao Chen, Jia-Cheng Huang, Meng-Xia Li, Le-Le Zhang, Da-Long Wan.

**Writing – review & editing:** Li Bao, ShengZhang Lin.
